# Visual SLAM-Based Robotic Mapping Method for Planetary Construction

**DOI:** 10.3390/s21227715

**Published:** 2021-11-19

**Authors:** Sungchul Hong, Antyanta Bangunharcana, Jae-Min Park, Minseong Choi, Hyu-Soung Shin

**Affiliations:** 1 Department of Geoinformatic Engineering, Inha University, Incheon 22212, Korea; jaemin@kict.re.kr; 2 Department of Mechanical Engineering, College of Engineering, Korea Advanced Institute of Science and Technology, Daejeon 34141, Korea; antabangun@kaist.ac.kr (A.B.); iphone7743@kaist.ac.kr (M.C.); 3 Department of Future Technology and Convergence Research, Korea Institute of Civil Engineering and Building Technology, Goyang 10223, Korea; hyushin@kict.re.kr

**Keywords:** planetary construction mapping, exploration rover, visual SLAM, deep learning, 3D terrain map

## Abstract

With the recent discovery of water-ice and lava tubes on the Moon and Mars along with the development of in-situ resource utilization (ISRU) technology, the recent planetary exploration has focused on rover (or lander)-based surface missions toward the base construction for long-term human exploration and habitation. However, a 3D terrain map, mostly based on orbiters’ terrain images, has insufficient resolutions for construction purposes. In this regard, this paper introduces the visual simultaneous localization and mapping (SLAM)-based robotic mapping method employing a stereo camera system on a rover. In the method, S-PTAM is utilized as a base framework, with which the disparity map from the self-supervised deep learning is combined to enhance the mapping capabilities under homogeneous and unstructured environments of planetary terrains. The overall performance of the proposed method was evaluated in the emulated planetary terrain and validated with potential results.

## 1. Introduction

The recent discovery of water-ice on the Moon and Mars has increased more potential for long-term human exploration and habitation, along with the technology development of in situ resource utilization (ISRU) [1,2,3,4]. ISRU refers to the generation of consumable products from raw materials on planetary surfaces, minimizing the dependence on Earth-based resources. For example, the water-ice can be utilized to produce O_2_ and H_2_O for life support or O_2_ and H_2_ for fuel and propellant [5,6]. Regolith, which is a major available resource, can be cold-pressed, sintered, and mixed with additives into a brick or a block as a construction material [7,8]. Large-scale additive manufacturing, formally known as 3D construction printing, is recognized as an efficient means of accumulating and aggregating the construction materials to provide quick and precise construction [9,10,11,12].

Unlike in planetary exploration, the planetary construction process indicates that humans and rovers will repeatedly visit the same location. Similarly to the robotic construction on Earth [13,14,15], various types of teleoperated and unmanned robots are expected to be employed in the planetary construction. A highly detailed and accurate 3D terrain map is essential for planning robotic operations to avoid obstacles and to construct a planetary infrastructure, or even a planetary base [16,17,18].

However, 3D terrain maps, which are mostly based on planetary orbiters, have insufficient spatial resolutions. The planetary surface has homogeneous and rough terrains in the absence of the global navigation satellite system (GNSS). Thus, for autonomous navigation and mapping, there have been active research efforts on 3D simultaneous localization and mapping (SLAM) techniques that employ a variety use of sensors including monocular or stereo camera, an RGB-D camera, and light detection and ranging (LiDAR). The SLAM technique enables a rover to estimate its current position and orientation as well as to construct a consistent map of its surrounding environments. For example, RGB-D and LiDAR SLAM utilize an active sensor technology that directly obtains a highly dense 3D point-cloud. However, a planetary rover’s capabilities are limited by communication links and bandwidth to Earth, a power supply, a computational resource, and a data storage capacity [19]. A monocular or stereo camera could be an alternative sensor due to its lighter weight and lower power consumption than those of the RGB-D camera and LiDAR. Also, 3D point-clouds with color information can be created, which are essential to remotely control a rover’s motion and operation as well as to identify a terrain feature on a planetary surface. A stereo camera can measure bearing and range avoiding a scale ambiguity problem from a monocular camera. In addition, the stereo camera system is mounted on recently designed rovers for the Moon and Mars explorations (e.g., Yutu rovers in Chang-E 3 and 4 missions and a Perseverance rover in the Mars 2020 mission) [20,21,22,23].

In this regard, a modern stereo SLAM is utilized to develop a robotic construction mapping method. However, there are technical limitations, such as the sparse nature of 3D point-clouds, a larger positional error from a longer distance measurement, and the difficulty of estimating a rover’s positions from homogeneous terrains. Thus, in this paper, the robotic mapping method, which is adaptive to the stereo SLAM, is proposed with enhanced capabilities to build a highly detailed and accurate 3D point-cloud for construction purposes.

## 2. Literature Review

### 2.1. Planetary Construction

**Figure 1:**
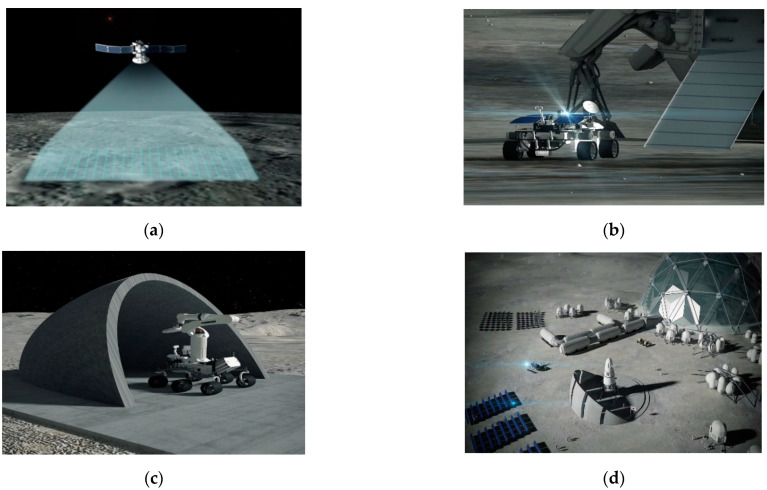
Conceptual process of a planetary construction: (**a**) construction site selection with a planetary orbiter; (**b**) construction site investigation with rover; (**c**) site preparation and infrastructure emplacement with a construction robot; (**d**) base and ISRU facility construction for human presence.

The international space community is becoming increasingly interested in and committed to robotic explorations toward development of infrastructure and a base on the Moon and, ultimately, to Mars [24]. The conceptual process on a planetary construction consists of a site selection, a site investigation, a site preparation and an infrastructure emplacement, and a base and ISRU facility construction (Figure 1).

Over the last few decades, planetary remote sensing data has been used to build global 3D terrain maps for landing site selection and path planning [25,26,27] and to assess the availability and distribution of in situ resources [28,29,30,31]. The remotely sensed data can also be utilized to locate the planetary infrastructure and base, meeting ISRU and civil engineering conditions to support a permanent and sustainable human presence on the Moon and Mars (Figure 1a) [32,33,34]. The ISRU conditions concern the ability to utilize indigenous materials, resources, and environments to extract useful commodities such as water, metals, and structural building materials. In the civil engineering conditions, the topographic and geotechnical feasibility to improve foundation and to stabilize surface are concerned, along with the development or maturation of engineering capability of robotic construction.

However, due to insufficient spatial resolutions of the remote sensing data, the robotic surface investigation is required to build the 3D terrain map with a high resolution and to characterize the geotechnical property and the resource availability on the construction site (Figure 1b). The 3D mapping result can then be used to plan a robotic construction operation for the site preparation. Ground excavation and obstacle clearing proceed to build a stable foundation for infrastructure emplacement [35,36] (Figure 1c). The planetary construction requires repeated visits to the same location by humans and robots. Therefore, a landing pad with a berm is a high initial priority to mitigate lander plume effects that can disturb regolith and damage human and robots. A road improves the mobility around the infrastructure and helps to minimize the maintenance of robots. Figure 1d shows the planetary base and ISRU facilities for a long-term human presence. In hostile environments of planetary surface, the lava tube is one of the potential candidate sites as it can maintain a stable temperature and provide protection from radiation and meteorites [4,37]. In the ISRU facilities, the highest priority is water production for life support and propellant production. Also, metal extraction is important for in-situ fabrication of spare devices and repairs [32].

### 2.2. Planetary SLAM

There have been numerous research efforts on planetary 3D SLAM techniques, in which the terrain perceptions and mapping results mainly depend on a sensor selection and a sensor fusion. In an early stage, the monocular SLAM frameworks were presented, involving the extended Kalman filter (EKF) and an improved feature detection and matching method to track unconstrained motion of a rover [38,39]. However, the Kalman filter only works well when distinct features are evenly distributed. Furthermore, using a single camera without inertial and range sensors causes scale ambiguity and the measurement drift. The RGB-D SLAM was developed as an alternative way of directly obtaining both depth per-pixel and visual texture information [40,41]. However, lighting circumstances can have a significant impact on the RGB-D camera, causing noisy and homogeneous point-clouds. Tong and Barfoot [17] proposed the LiDAR SLAM for a future lunar base. In this research, the global terrain map was made with a sparse-feature-based method and a batch alignment algorithm that solve the robustness problems of feature association and measurement outliers. In addition, Shaukat et al. [42] presented the camera-LiDAR fusion SLAM that takes advantages of either of these individual sensors. Specifically, LiDAR overcomes the field of view and point density limitations of a camera. However, dense 3D point-clouds from LiDAR affect a significant computation cost and a large data storage. Also, when considering that a rover with a solar array has a limited power, LiDAR sacrifices other scientific and civil engineering payloads to investigate in-situ resources and underground structural properties. Carrio et al. [43] proposed the stereo-inertial SLAM based upon a stereo camera pair, inertial measurement unit (IMU), and wheel odometer. A terrain map was created through multisensor fusion, and its accuracy was improved by predicting non-systematic errors from the wheel interaction with the Gaussian-based odometer error model. Also, Schuster et al. [19] presented the lighter rover unit (LRU), in which the field programmable gate array (FPGA) board for a stereo dense mapping aids the localization and mapping process in combination with the IMU and wheel encoder. In the research, the exploration rover was designed to traverse longer with low-level supervision. The stereo-inertial SLAM requires a high-level of onboard autonomy, and all computation should be conducted on board.

In this paper, the stereo SLAM for construction mapping is under consideration. In comparison with the exploration rovers, the construction mapping rover is expected to traverse a limited extent of construction candidate sites where terrains are relatively flat with a sparse rock distribution. In addition, a more generic approach is preferred in that a stereo-camera is one of the compulsory payloads for current and future rovers. For which, S-PTAM [44], which is a modern stereo SLAM, is adopted and further modified for planetary construction mapping. S-PTAM estimates the camera pose by matching the correspondences between a terrain feature and identical features on the stereo image. Once the current pose is estimated, the next image pair is selected as a key frame if the feature match is lower than a predefined threshold (e.g., 90%) of the feature match in the last keyframe. When the threshold is satisfied, the remaining unmatched image features are triangulated with terrain features and added to the camera pose. Those of the keyframe are stored in the stereo keyframe database. The bundle adjustment, which involves a series of keyframes from the beginning, is used to locally optimize the camera poses. This procedure is repeatedly performed to localize the camera.

The construction mapping requires a rover to move around a large extent of unknown environments. The recognition of already-visited places is required to globally optimize the camera trajectory. When the loop-closing is detected using the Bag of Words (BoW) [45], the camera trajectory is then optimized using the graph optimization process that minimizes the accumulated drift and consequently maintains a globally consistent trajectory of the camera pose. However, the sparse nature of unevenly distributed point-clouds makes them insufficient for construction mapping. The high-resolution terrain map is essential for a construction robot to identify an obstacle distribution and to stabilize a construction site. In this regard, the novel dense-mapping method is proposed to enhance the mapping capability, which is described in Section 3.

## 3. Proposed Method

### 3.1. System Architecture

**Figure 2:**
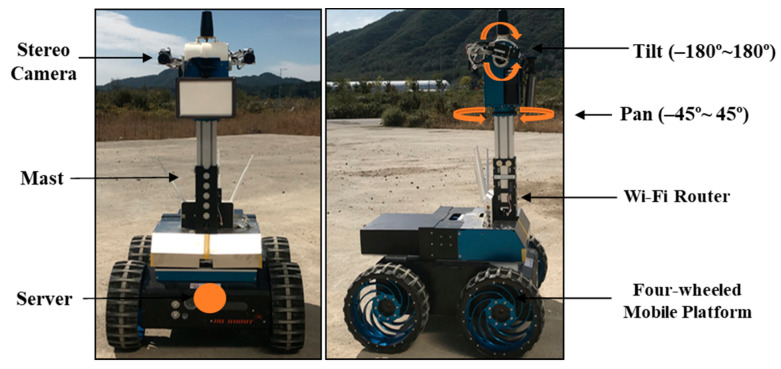
Overview of a robotic mapping system.

The robotic mapping system is designed to simulate a planetary construction mapping process in an exploration manner (Figure 2). The rover is a four-wheeled mobile platform with payloads consisting of a Wi-Fi enabled router and a stereo camera mounted to the top of pan-tilt mast. The stereo camera setup has a baseline of 20 cm with a resolution of 484 × 366 pixels per image. The pan and tilt units are capable of 360 degrees and 90 degrees of command motions in horizontal and vertical directions, respectively. The stereo camera system enables the rover to effectively collect terrain images, minimizing the rover’s motions. The router allows the remote computer to control the movements of the rover’s camera system and four wheels. Also, terrain images can be transmitted to the remote computer for a dense 3D point-cloud mapping.

### 3.2. Stereo SLAM-Based 3D Mapping Method

**Figure 3:**
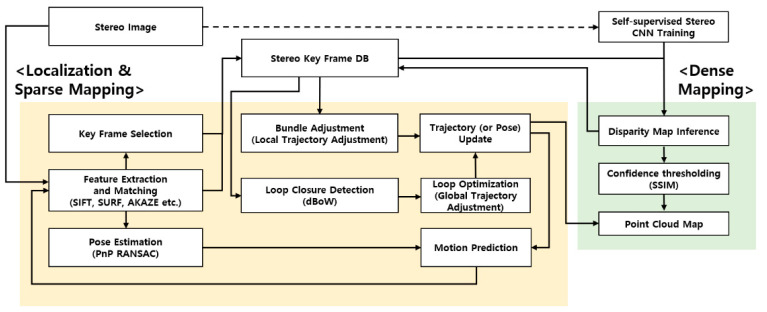
Overview of the proposed method.

This section describes the visual SLAM-based robotic mapping method for the planetary construction sites. Figure 3 shows the overall flow of the proposed method consisting of two main threads: mapping and localization. In the beginning, a series of stereo image pairs from the camera are used to train the self-supervised deep-learning model, from which the disparity map is then estimated (Section 3.2.1). The disparity map is used to create the dense 3D point-cloud map in the mapping thread (Section 3.2.2), and also to improve the robustness of a rover trajectory in the localization thread (Section 3.2.3).

#### 3.2.1. Disparity Map Prediction

**Figure 4:**
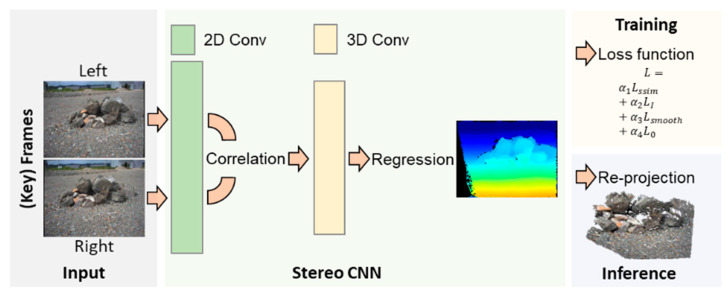
Self-supervised stereo CNN for dense point-cloud mapping.

Recently, the deep-learning-based approach towards the dense stereo disparity prediction [46,47,48,49] has been shown to outperform traditional methods [50] in multiple benchmarks [46,51,52]. However, the training dataset available on Earth is not practical for planetary terrains. To build an adaptable deep-learning model without the need of building a ground-truth dataset, the self-supervised deep-learning model [53] is adopted and modified for the planetary construction mapping. The overall training and inference flows are shown in Figure 4, in which a dataset of planetary terrain images is collected from the stereo cameras on the rover. A subset of the collected images is involved for training the deep-learning model that consists of 2D and 3D convolutional layers based on geometric-based CNN (convolutional neural network) models [54]. The 2D convolution layers, which are used to extract image features from each image, construct a cost volume. In the 3D convolution layers, the cost volumes are aggregated to infer disparity values using the probability distribution at each pixel. The disparity map is eventually constructed as a regressed from of the probability distribution.

In the training (Figure 4), the loss function ($L\left(I_{L},I_{R\to L},D_{L}\right)$) is computed with the disparity map and the stereo image in Equation (1), where $I_{L}$ is the left image and $I_{R\to L}$ is the right image shifted using the predicted disparity map denoted as $D_{L}$.

(1) $$L\left(I_{L},I_{R\to L},D_{L}\right)=\alpha_{1}L_{SSIM}\left(I_{L},I_{R\to L}\right)+\alpha_{2}L_{I}\left(I_{L},I_{R\to L}\right)+\alpha_{3}L_{Smooth}\left(I_{L},D_{L}\right)+\alpha_{4}L_{0}\left(D_{L}\right)\;$$

The structural similarity loss $\left(L_{SSIM}\left(I_{L},I_{R\to L}\right)\right)$ is used to penalize the structural difference between the left image and the reconstructed left image (Equation (2)), where $SSIM\left(I_{L},I_{R\to L}\right)$ is the structural similarity between the left and right images.

(2) $$L_{SSIM}\left(I_{L},I_{R\to L}\right)=\frac{1}{N}\sum^{\;}\frac{1-SSIM\left(I_{L},I_{R\to L}\right)}{2}$$

The RGB difference between the left and right images at each pixel, which is denoted as $L_{I}\left(I_{L},I_{R\to L}\right)$, is also computed as follows:

(3) $$L_{I}\left(I_{L},I_{R\to L}\right)=\frac{1}{N}\sum^{\;}\left|I_{L}-I_{R\to L}\right|$$

The smoothness loss ($L_{Smooth}\left(I_{L},D_{L}\right))$ regularizes the predicted disparity to be smooth in Equation (4), where $\nabla_{x}$ and $\nabla_{y}$ denotes the gradient operators for *x* and *y* directions, respectively.

(4) $$L_{I}\left(I_{L},I_{R\to L}\right)=\frac{1}{N}\sum^{\;}\left|I_{L}-I_{R\to L}\right|$$

In Equation (5), the sum of all the disparities is minimized to regularize the images, especially for the homogeneous terrains on the planetary surface.

(5) $$L_{0}\left(D_{L}\right)=\frac{1}{N}\sum^{\;}\left|D_{L}\right|$$

In the inference (Figure 4), to avoid erroneous disparity predictions, the structural difference between the left and right images in Equation (2) is repeatedly computed at each pixel. The pixels with the structural difference less than a predefined threshold are not involved in the dense point-cloud mapping. In Equation (6), 3D points are reconstructed from the disparity map as follows:

(6) $$\left[\begin{array}{c} \hat{X_{3d}}\\ \hat{Y_{3d}}\\ \;\hat{Z} \end{array}\right]=K^{-1}\;\left[\begin{array}{c} x_{2d}\;\cdot \;\hat{Z}\\ y_{2d}\cdot \;\hat{Z}\\ \;\hat{Z} \end{array}\right]\hat{Z}=fb/D_{L}\left[x_{2d},y_{2d}\right]$$

where K, f, and b are the intrinsic matrix, the focal length, and the baseline of the camera obtained by camera calibration respectively. $D_{L}\left[x_{2d},y_{2d}\right]$ is the estimated disparity at 2D coordinate $\left(x_{2d},y_{2d}\right)$. To limit computation cost, the dense disparity map is only computed at the keyframes instead of at every frame.

#### 3.2.2. Disparity Map for 3D Mapping

The disparity map is a basis of creating a 3D dense point-cloud in the mapping module (Figure 3). However, a 3D dense point-cloud at each keyframe is referenced at the local coordinate system as described in Section 3.2.1. To combine point-clouds at each own local coordinate system, the coordinate transformation is required to reference all point-clouds at the global coordinate system as follows:

(7) $$\left[\begin{array}{c} X_{3d}\\ Y_{3d}\\ Z \end{array}\right]=R\left[\begin{array}{c} \hat{X_{3d}}\\ \hat{Y_{3d}}\\ \hat{Z} \end{array}\right]+t$$

where $R\in {\mathbb{R}}^{3\times 3}$ and $t\in {\mathbb{R}}^{3}$ are the rotation and translation matrix with respect to the global coordinate system. When all points are re-projected at the identical coordinate system, points from different keyframes can have corresponding positions. Thus, the voxel (or 3D grid) is used to register and handle 3D points in an identical space at the predefined resolution. Each voxel contains the coordinate and the RGB color property of a 3D point. When multiple points are registered into the same voxel, the voxel property is updated accordingly. In the dense 3D mapping, the voxel can decrease the number of 3D points with the predefined resolution as well as to remove noisy 3D points when the number of points is less than predefined number.

#### 3.2.3. Disparity Map for Localization

In the localization module, the disparity map is used to improve S-PTAM, mainly for the trajectory and the loop closure shown in Figure 3. In the rover trajectory, the increased number of feature points from each frame can be reliably tracked from the reference keyframe, influencing the improvement of the localization accuracy.

In the feature-matching procedure, the nearest neighbor distance ratio (NNDR) constraint is typically employed to accept point matches if the distance ratio between the first- and second-best matches is below a predefined threshold. However, when two matches are too ambiguous to be discriminated, the NNDR constraint can lead to the loss of important feature points. Thus, the disparity map is utilized as an additional constraint to increase the number of feature point matches concerning the homogeneous planetary terrains. Given a stereo keyframe, the nearest neighbor of the feature matches is first obtained, following the epipolar line. The disparity between two points is then computed and compared with the corresponding position on the disparity map. The difference between the two disparities, which is lower than the threshold, is another constraint to accept the feature matches. In practice, the tracking procedure uses the point matches that satisfy the NNDR or the disparity constraint thresholds.

In addition, when the rover returns to the previously visited place, the loop closure globally minimizes the accumulated positional errors of the rover trajectory. However, an incorrect loop detection can lead to erroneous results of both localization and mapping. To increase the reliability, 3D points from the disparity map are used to geometrically verify the loop closure rather than the sparse 3D points from stereo correspondence. The increased number of 3D points from the disparity map can improve the success rate of true loop closure.

## 4. Experiments and Results

### 4.1. Overview

**Figure 5:**
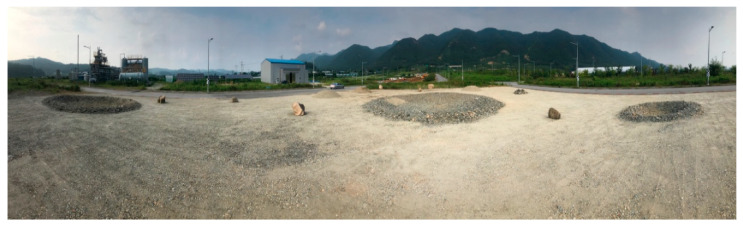
Emulated planetary terrain at KICT.

The rover depicted in Figure 2 was deployed to obtain terrain images at the test site in the Korea Research Institute of Civil Engineering and Building Technology (KICT) (Figure 5). The test site is the emulated planetary terrains of 40 m × 50 m where gravel is distributed over a flat ground with rocks, craters, and mounds. In the robotic mapping operation, the stereo camera was set up with the right (+90 degrees) or left (–90 degrees) direction rather than the front direction (0 degrees). As a result, the rover can efficiently collect terrain images while moving forward. Also, the stereo camera was fully rotated at periodic stops around a terrain feature. The proposed method was then applied to terrain images for creating a 3D point-cloud map that is converted to a 3D terrain map such as DEM (digital elevation model) and hillshade.

The purpose of these experiments is to evaluate capabilities of the proposed method in the emulated planetary terrains. In Section 4.2, the disparity map based on the stereo CNN model was verified concerning its impacts on the mapping and localization capabilities of the proposed method. The 3D dense point-cloud as a mapping result was evaluated with the terrestrial LiDAR data and was converted to 3D terrain maps for demonstration purposes in Section 4.3.

### 4.2. Parameter Setting in the Proposed Method

The dense disparity prediction model is closely related to the localization and mapping modules in the proposed method. The experiment and verification of the stereo CNN model as well as its impact on feature matching and dense mapping are presented in this section.

#### 4.2.1. Dense Disparity Estimation

The self-supervised stereo CNN model is first trained on the publicly available SceneFlow dataset [46], consisting of 35,454 synthetic training stereo images and ground truth depth. The model is trained for 10 epochs with a learning rate of 0.001 for the first 7 epochs and 0.0001 for the remaining epochs. An Adam optimizer was used with $\beta_{1}=0.9,\beta_{2}=0.999$. Augmentation was conducted by cropping the images into size of 512 × 256. As the ground true depth is available for the training, the smooth loss is used as follows:

(8) $$Smooth_{L_{1}}=\{\begin{array}{c} 0.5x^{2},\;\;\;\;\;\;\;\;\;\;\;\;\;\;if\;\left|x\right|<1\;\\ \left|x\right|-0.5,\;\;\;\;\;\;\;\;\;otherwise\; \end{array}$$

The stereo CNN model is further finetuned using the collected terrain images from the test site in Figure 5. The learning rate for finetuning is set at 0.0001 and trained for only 1 epoch with a dataset size of around 20,000 images. During the training, the images are randomly cropped into a size of 320×384. Further image augmentations in the form of random Gaussian noise, random brightness and contrast change, and random shift of each RGB channel, are applied to obtain a more generalized model. The loss function constants in Equation (1) are set at $\alpha_{1}=0.8,\alpha_{2}=0.2,\alpha_{3}=0.1,\alpha_{4}=0.1$.

**Figure 6:**
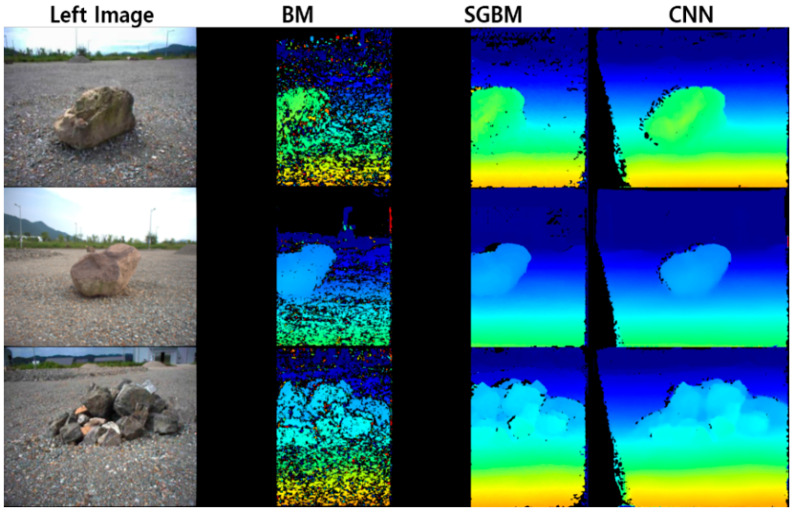
Comparison of dense disparity map.

The stereo CNN model, designed to run on the RTX 2080Ti GPU, is able to run at around 20 ms per stereo frame with a resolution of 366×484 from the rover. In the practice, pixels with $L_{SSIM}\left(I_{L},I_{R\to L}\right)$ values less than 0.4 are rejected and shown in black. In the other words, a black pixel indicates that prediction is made inaccurately. In Figure 6, the stereo CNN model is qualitatively compared to other traditional methods including block matching (BM) and semi-global block matching (SGBM). Although a set of parameters is carefully determined, the BM-based disparity map contains much noise comparing to the disparity maps from SGBM and CNN. The BM and SGBM only describe disparities about an overlapped region of the stereo image. However, the stereo CNN predicts disparities more than the overlapped region, minimizing the number of black pixels. The experiment results show that the predicted disparity map from the stereo CNN model is able to build denser 3D point-clouds, reducing the rover’s motions.

#### 4.2.2. Disparity-Map-Aided Feature Matching

**Figure 7:**
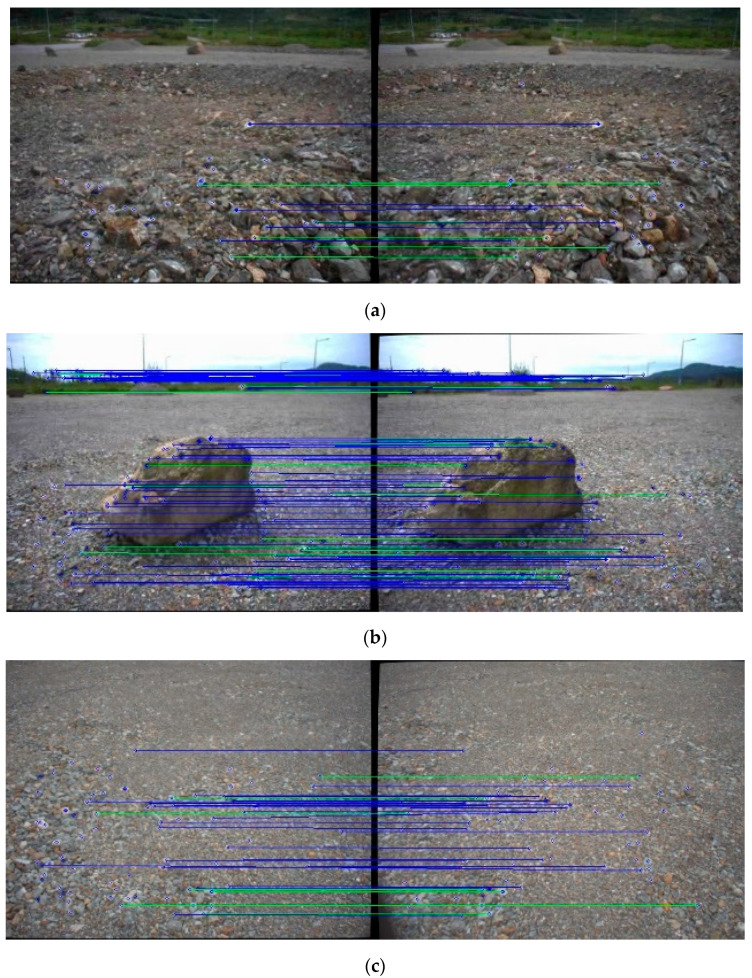
Feature-matching results from terrain features with AKAZE (threshold) (blue lines: feature match by disparity map and NNDR constraints, green lines: a feature match by disparity map but not NNDR constraint). (**a**) Crater with AKAZE (0.005); (**b**) rock with AKAZE (0.002); (**c**) ground with AKAZE (0.002).

The number of feature matches from the stereo camera is closely related to the accuracy of rover trajectory. In the proposed method, the disparity map predicted by the stereo CNN model is used as additional constraint to increase feature matches from a stereo image pair. AKAZE [55] is selected to extract and match feature points due to its prompt and robust performance on planetary terrain images [56] and the test site in Figure 5. In the experiment, AKAZE with multiple thresholds is applied to three different types of terrain features including the ground, rock, and craters. Feature matches using the brute-force method are then filtered with the NNDR with the distance ratio of 0.6 and the disparity constraint with the difference ratio of 3. The average of feature matches is categorized as the relative complement of the disparity map constraint in the NNDR constraint $\left(\mathrm{NNDR}\cap^{\;}{\mathrm{Disparity}}^{c}\right)$, the intersection of the NNDR and disparity map constraints $\left(\mathrm{NNDR}\cap^{\;}\mathrm{Disparity}\right)$, and the relative complement of the NNDR constraint in the disparity map constraint $\left({\mathrm{NNDR}}^{c}\cap^{\;}\mathrm{Disparity}\right).$ Table 1 shows that the matching results by the NNDR, excluding disparity map constraint, are very few. Most matched features are identical to the matched results from the disparity map constraint. Nevertheless, the disparity map constraint yields more accepted feature matches in comparison to the NNDAR constraint, which indicates that the dense map constraint does not miss many of the matches accepted by NNDR and accepts the matches missed by NNDR. In Figure 7, AKAZE with large thresholds is arbitrarily applied for visual analysis. Blue lines are feature matches accepted by both constraints, and green lines are feature matches accepted by the disparity map but not the NNDR constraint. Feature-matching results confirm that all of the green and blue lines are correctly matched, which indicates that the disparity map constraint in each keyframe increases the number of extracted map points and may improve the robustness of the localization module in the proposed method.

### 4.3. Terrain-Mapping Results

A 3D terrain map derived from the high-resolution terrain imagery of the Moon’s and Mars’ orbiters depicts a general representation of terrain features. However, for construction purposes, the terrain map has an insufficient resolution to clearly identify the size and distribution of obstacles as well as to accurately compute an earthwork volume of craters and mound. Thus, in the experiment, the rover traversed the entire test site for a detailed 3D reconstruction of the emulated planetary terrain.

**Figure 8:**
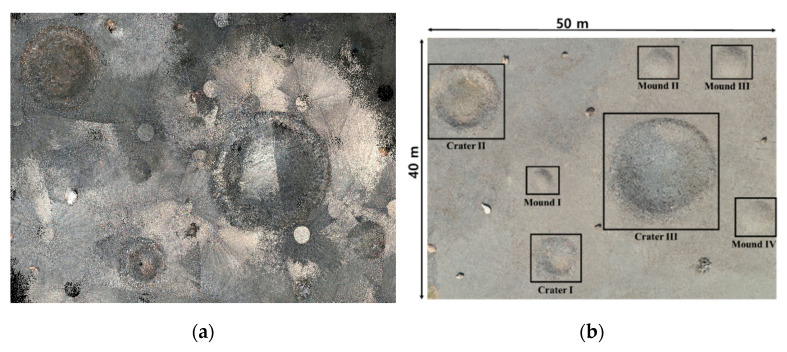
The 3D point-cloud mapping results and terrain features in the test site: (**a**) 3D dense point-cloud; (**b**) terrain features.

**Figure 9:**
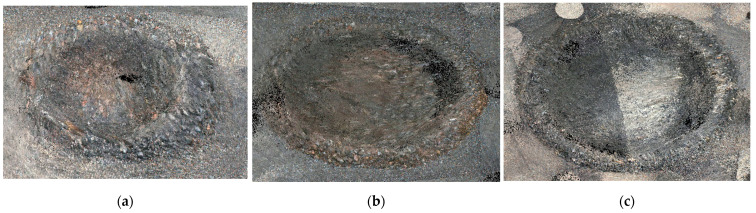

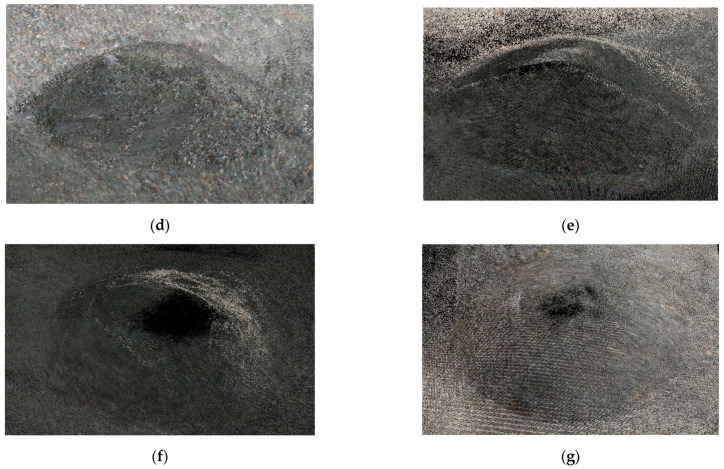
The 3D dense point-clouds of terrain features: (**a**) crater I; (**b**) crater II; (**c**) crater III; (**d**) mound I; (**e**) mound II; (**f**) mound III; (**g**) mound IV.

In the robotic mapping, the disparity map computed from stereo images estimates the depth per pixel. The depth value combined with the color information is used create a colored point-cloud. The rover operation in the test site imposed sensing restrictions due to variant lighting conditions and rugged terrains. All terrain images were collected in daytime. However, changes in weather and solar altitude led point-clouds to have inconsistent color properties, making it difficult to identify the morphological properties of terrain features (Figure 8a). Figure 9 shows point-clouds of craters and mounds, each of which is indexed in Figure 8b. The crater and mound contain bright and consistent colors of point-clouds in Figure 9a,g. However, the atmospheric clouds decreased the brightness of terrain images, and the point-clouds have dark colors in Figure 9b,d. Partial and moving atmospheric clouds continually changed illumination conditions, and the point-cloud in Figure 9c consequently has a mixture of beige and gray colors. Also, the multiple circles were created when the stereo camera was fully rotated in horizontal direction. The point-clouds in Figure 9e,f are dark gray due to the low solar altitude in the late afternoon. In addition, the mounds, shown in Figure 9e–g, have empty point-clouds on top as they are higher than the stereo camera mounted on the rover mast.

**Figure 10:**
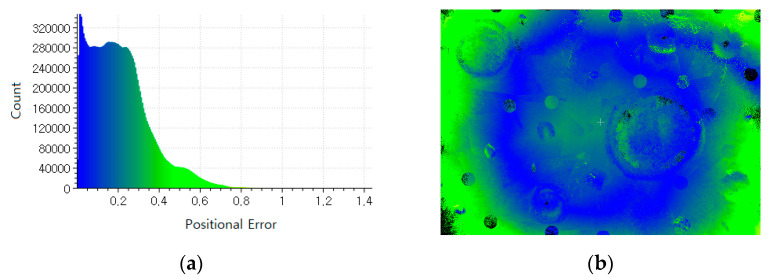
Positional error assessment to measured point-clouds (identical magnitude of a positional error from blue to yellow-green in both sub-figures): (**a**) positional error histogram; (**b**) positional error distribution.

The terrestrial LiDAR was used to create the reference point-cloud to evaluate the quality of the 3D point-cloud derived from the proposed method. The two sets of point-clouds were optimally aligned by iterative closest point (ICP). The root mean Square Error (RMSE) was then computed as 0.27 m. Also, in Figure 10, the positional error distribution is computed along with the positional error histogram. The RMSE result shows a good indication of the overall performance of the proposed method. However, the positional errors are not evenly distributed over the test site in Figure 10b, where the positional errors in the marginal area are larger, in that the rover mainly traversed around the middle area of the test site. In Figure 10a, the positional error histogram shows that more than 95% of positional errors are within 0.5 m.

**Figure 11:**
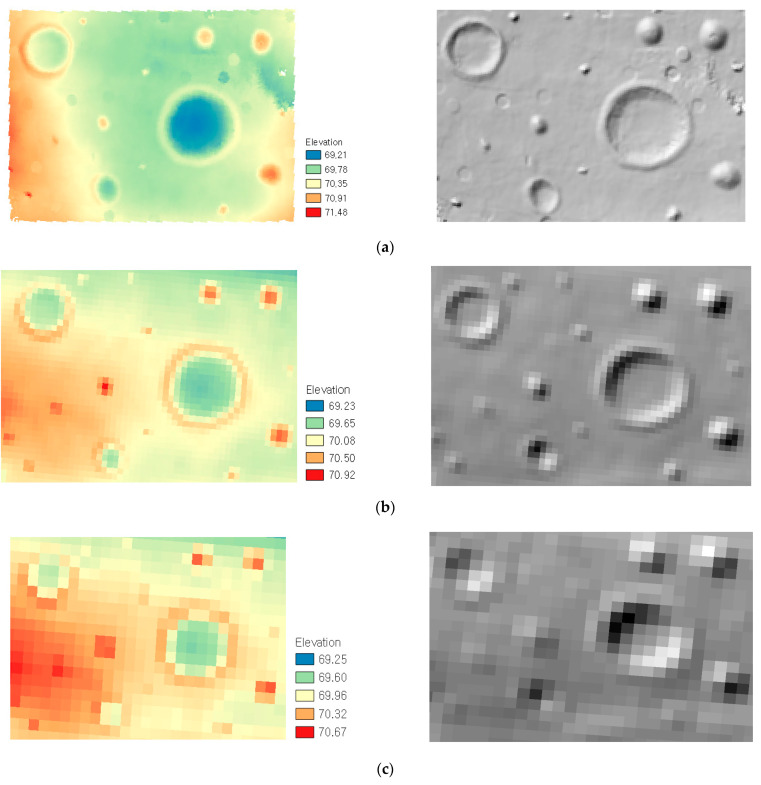
The 3D terrain maps (resolution): (**a**) measured DEM and hillshade (0.3 m); (**b**) emulated DEM and hillshade (1 m); (**c**) emulated DEM and hillshade (2 m).

The proposed method aims to build a 3D terrain map for construction purposes. The DEM and hillshade with 0.3 m resolution were created concerning the accuracy and density of the measured point-clouds (Figure 11a). Also, for comparison purposes, the emulated DEM and hillshade of 1 m and 2 m resolutions, shown in Figure 11b,c, respectively, were created from the reference point-cloud. Figure 11 shows that all 3D terrain maps depict the distribution of major terrain features (e.g., crater and mound). However, the size and shape of the terrain features become distorted and exaggerated as the resolution of 3D terrain maps decreases. For example, the emulated train maps in Figure 11b,c have the larger grid size than obstacle (e.g., a rock and stone pile). The types of an obstacle are not clearly distinguished from each other. Figure 11a shows that the 3D terrain map from the proposed method has sufficient spatial resolution to show the obstacle distribution as well as to clearly identify the shape of craters and mound for the construction planning (e.g., path planning and obstacle clearing) and design (e.g., infrastructure emplacement) purposes.

## 5. Summary and Conclusions

The recent discovery of water-ice and lava tubes from the Moon and Mars has facilitated new ideas and proposals to construct a base station for the long-term human exploration and habitation. Meanwhile, the ISRU technology provides a means of substantially reducing the cost and mass of resources that must be launched from Earth. In the planetary construction, humans and rovers are required to repeatedly visit the same location to build an infrastructure and a base station. The 3D terrain map with high resolution is essential for the construction design and the construction robot operation. For which, a robotic mapping method should be employed, as 3D terrain maps mostly derived from an orbiter’s terrain images have insufficient resolutions. However, the planetary surface has homogeneous and unstructured terrains under a GNSS-denied environment.

In this regard, this paper presents the visual SLAM-based robotic mapping method for planetary construction. The proposed method combines the stereo SLAM with the deep-learning-based stereo dense matching method to produce a highly detailed 3D point-cloud in unknown planetary environments. Specifically, S-PTAM is adopted as a base stereo SLAM framework. However, due to its sparse nature of unevenly distributed point-clouds, the self-supervised stereo CNN model for disparity map estimation is used to enhance the mapping capabilities. The major innovations of the proposed method are as follows: first, the stereo CNN model is able to build the disparity map without the ground-true disparity maps of a planetary terrain. Second, the disparity map prediction is used to enrich point-clouds more than the stereo image overlapped region. A high-resolution 3D terrain map can be constructed minimizing the rover’s motions. Third, the disparity map is also utilized as an additional constraint to increase the number of point-clouds for tracking and loop closing. The proposed method was applied to the emulated planetary terrain and evaluated with point-clouds from the terrestrial LiDAR. Experiment results confirmed that the stereo camera system on the rover can create highly detailed 3D point-clouds to build a 3D terrain map under homogeneous and unstructured environments.

Although the proposed method shows the potential use for planetary exploration and construction, the camera system on the rover is vulnerable to variant illumination conditions. The degraded images reveal limitations to visually identify and analyze terrain features, and thereby the image enhancement method will be definitely worth investigating to restore the visibility, color, and natural appearance of planetary terrain features. In addition, although the proposed method is effective for a local 3D terrain mapping, the rover trajectory is relatively referenced to its own coordinate system. The global localization is another concern to correct the estimate of a rover trajectory and to align a rover-based 3D local map to an orbiter-based 3D global map.

## Figures and Tables

**Table 1:** Number of feature matches with different thresholds in AKAZE.

Thresholds	Number of Feature Detected	Matching Constraint		
		$\mathrm{NNDR}\cap^{}{\mathrm{Disparity}}^{c}$	$\mathrm{NNDR}\cap^{}\mathrm{Disparity}$	$NNDR^{c}\cap^{}Disparity$
0.001	337	2	133	58
0.0001	1758	9	589	329
0.00001	2983	3	898	603
